# Associations of corin genetic polymorphisms with salt sensitivity, blood pressure changes, and hypertension incidence in Chinese adults

**DOI:** 10.1111/jch.14401

**Published:** 2021-11-30

**Authors:** Ting Zou, Shi Yao, Ming‐Fei Du, Jian‐Jun Mu, Chao Chu, Gui‐Lin Hu, Yue‐Yuan Liao, Chen Chen, Dan Wang, Qiong Ma, Yu Yan, Hao Jia, Ke‐Ke Wang, Yue Sun, Ze‐Jiaxin Niu, Xi Zhang, Rui‐Chen Yan, Zi‐Yue Man, Dan‐Feng Ren, Lan Wang, Wei‐Hua Gao, Hao Li, Yong‐Xing Wu, Chun‐Hua Li, Ke Gao, Jie Zhang, Tie‐Lin Yang, Yang Wang

**Affiliations:** ^1^ Department of Cardiovascular Medicine First Affiliated Hospital of Xi'an Jiaotong University Xi'an China; ^2^ Key Laboratory of Molecular Cardiology of Shaanxi Province Xi'an China; ^3^ National and Local Joint Engineering Research Center of Biodiagnosis and Biotherapy Second Affiliated Hospital of Xi'an Jiaotong University Xi'an China; ^4^ Department of Infectious Diseases the First Affiliated Hospital of Xi'an Jiaotong University Xi'an China; ^5^ Department of Cardiology Xi'an International Medical Center Hospital Xi'an China; ^6^ Department of Cardiology Xi'an No.1 Hospital Xi'an China; ^7^ Department of Critical Care Medicine First Affiliated Hospital of Xi'an Jiaotong University Xi'an China; ^8^ Department of Ophthalmology Xi'an People's Hospital Xi'an China; ^9^ Department of Cardiology Xi'an People's Hospital Xi'an China; ^10^ Biomedical Informatics & Genomics Center School of Life Science and Technology Xi'an Jiaotong University Key Laboratory of Biomedical Information Engineering of Ministry of Education Xi'an China

**Keywords:** blood pressure, corin, gene polymorphism, salt sensitivity, salt

## Abstract

Corin, a transmembrane serine protease that can cleave pro‐atrial natriuretic peptide (Pro‐ANP) into smaller bioactive molecule atrial natriuretic peptide, has been shown to be involved in the pathophysiology of hypertension, cardiac hypertrophy. We sought to examine the associations of corin genetic variations with salt sensitivity, blood pressure (BP) changes and hypertension incidence. We studied participants of the original Baoji Salt‐Sensitive cohort, recruited from 124 families from seven Chinese villages in 2004 who sequentially received a usual baseline salt diet, a 7‐day low salt diet (3 g/day) and a 7‐day high salt diet (18 g/day), respectively. They were followed up for 8 years (in 2009, 2012) to evaluate the development of hypertension. Corin SNP rs3749584 was significantly associated with diastolic BP (DBP) and mean arterial pressure (MAP) response to low‐salt diet, while rs4695253, rs17654278 were associated with pulse pressure (PP) response to low‐salt diet. SNPs rs4695253, rs12509275, rs2351783, rs2271036, rs2271037 were significantly associated with systolic BP (SBP), DBP, and MAP responses to high‐salt diet. In addition, SNPs rs12641823, rs6834933, rs2271036, and rs22710367 were significantly associated with the longitudinal changes in SBP, DBP, MAP, or PP over 8 years of follow‐up. SNP rs73814824 was significantly associated with the incidence of hypertension over 8 years. Gene‐based analysis showed that corin gene was significantly associated with longitudinal BP changes and hypertension incidence after 8‐year follow‐up. This study suggests that corin may play a role in salt sensitivity, BP progression, and development of hypertension.

## 1 INTRODUCTION

Hypertension is determined by environmental factors, genetic factors, and their interactions.^1^ Among the environmental determinants of blood pressure (BP) variation, high‐dietary salt intake seems to be the most significant.^2^ However, BP response to dietary salt intake varies considerably among individuals, a phenomenon known as salt sensitivity.^3^ Epidemiological data suggest that individual genetic map may play a crucial role in determining individual BP response to salt intake changes.^4^, ^5^, ^6^, ^7^ Therefore, the identification of genetic variants related to salt sensitivity would enhance our understanding of biological mechanisms of BP regulation.

Corin is a transmembrane serine protease of the trypsin superfamily, which can convert pro‐atrial natriuretic peptide (Pro‐ANP) into atrial natriuretic peptide (ANP) with biological activity.^8^ It is mainly expressed in myocardial cells, and also found in kidneys and uterus.^9^ In mice, corin deficiency prevents the transformation of Pro‐ANP to ANP, leading to hypertension and cardiac hypertrophy.^10^ Although the etiology of salt‐sensitive hypertension is undoubtedly multifactorial, there is experimental evidence linking corin to the pathogenesis of salt‐sensitive hypertension. Wang et al.^11^, ^12^ found that corin knockout mice exhibited reduced sodium excretion and impaired natriuretic peptide processing, which maybe an important mechanism in salt‐sensitive hypertension. However, the associations of corin with salt sensitivity of BP in humans have not been studied previously.

The human corin gene is composed of 22 exons with a total length of about 200 KB on chromosome 4.^13^ Prior studies showed that corin genetic variants were associated with hypertension in African Americans.^14^, ^15^, ^16^, ^17^ Mutations that reduce corin activity were also found in families of patients with hypertension and preeclampsia.^18^ However, none of previous studies have fully considered the gene–environment interactions on BP, particularly regarding dietary salt intake. Failure to measure such gene–environment interactions may obscure the genetic contribution to BP variability. Furthermore, no study has yet examined whether genetic variants in the corin gene can predict BP changes or the development of hypertension over time.

In the present study, we aimed to determine the relationships between genetic variations in the corin gene and BP response to strict dietary salt intervention in our previously established cohort. We also used both single marker‐based and gene‐based analyses to examine the associations of corin gene with longitudinal BP changes and hypertension incidence.

## 2 METHODS

### 2.1 Study participants

This was a retrospective cohort study consisted of 514 adults from 124 families in seven villages in Baoji City, Shaanxi Province, China, from April to November 2004. Both two‐generation (probands, their parents and siblings) and three‐generation (spouses and offspring of probands) families were recruited in this study. The detailed study design has been published previously.^6^, ^19^, ^20^, ^21^ Probands, their siblings, spouses and offspring participated in the chronic salt intake intervention in 2004, which has been described previously.^22^, ^23^, ^24^, ^25^ Briefly, the protocol comprised a questionnaire survey and physical examination during a 3‐day baseline observation period, a low‐salt diet for 7 days (3 g of salt or 51.3 mmol of sodium per day), and a high‐salt diet for additional 7 days (18 g of salt or 307.8 mmol of sodium per day). Dietary potassium intake remained unchanged during the two intervention phases.

**FIGURE 1:**
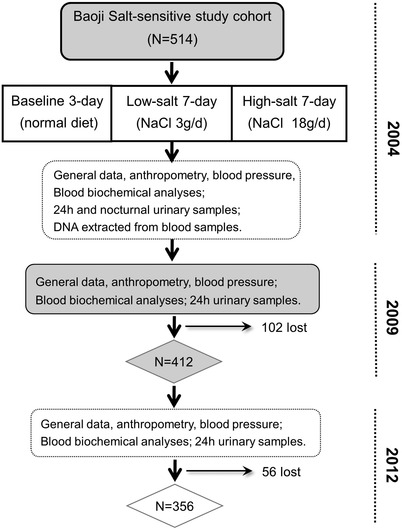
The protocol of Baoji Salt‐sensitive Cohort Study

To identify the associations of potential genetic polymorphisms with longitudinal BP change and hypertension incidence, we followed up this cohort in 2009 and 2012, and a total of 356 were followed up in 2012. Among the 514 eligible subjects, 102 were lost to follow‐up in 2009 and 56 were lost in 2012 (Figure 1). In the follow‐up evaluations, a 3‐day examination was performed as in 2004. Data on the hypertension history and the use of antihypertensive drugs was obtained using a standard questionnaire. Three BP measurements were obtained during each of the 3‐day follow‐up visits. The mean of the nine BP measurements was used for analysis.

The protocol was approved by the Ethics Committee of First Affiliated Hospital of Xi'an Jiaotong University (code: 2015–128). Written informed consents for the baseline examination and the intervention protocol were obtained from each participant. This study follows the principles of the Helsinki declaration, and all procedures were strictly implemented in accordance with institutional guidelines (ClinicalTrials.gov. registration number: NCT02734472).

### 2.2 BP measurement and definition of BP response to dietary intervention

BP was measured with a standard mercury sphygmomanometer in the sitting position as previously described.^26^, ^27^, ^28^ BP was measured by trained and certified observers during the 3‐day baseline observation period and on days 5, 6, and 7of each of the two 7‐day intervention periods. Hypertension was defined as systolic BP (SBP) ≥ 140 mmHg, diastolic BP (DBP) ≥ 90 mmHg or use of antihypertensive drugs according to participants’ clinical data or self‐report.^29^ The mean arterial pressure (MAP) was defined as SBP + (2 × DBP). Pulse pressure (PP) was calculated as SBP – DBP.

BP changes from the low‐salt intervention to the high‐salt intervention may provide a more valid phenotype measure for salt sensitivity as participants’ salt intake was controlled during both phases. On the other hand, identifying genetic determinants of BP response to a low‐salt diet from a usual diet should have more direct clinical and public health implications. Therefore, as described previously, we defined BP responses as follows: BP response to high salt = BP on the high‐salt diet – BP on the low‐salt diet; and BP response to the low‐salt = BP on the low‐salt diet – BP at baseline.^5^, ^6^, ^7^, ^19^

### 2.3 Blood and urinary biochemical analyses

The total cholesterol, triglycerides, high‐density lipoprotein (HDL) and fasting glucose levels, urinary concentrations of sodium and potassium were measured by automatic biochemical analyzer (Hitachi, Tokyo, Japan) as previously described.^5^, ^7^, ^30^, ^31^ The 24‐h urine samples were collected at baseline and on day 7 of each intervention to ensure the subjects’ compliance with the chronic salt intervention protocol. The 24‐h urinary excretions of sodium and potassium were quantified by multiplying the concentrations of sodium and potassium, respectively, by the total 24‐h urine volume.

### 2.4 SNP selection and genotyping

Fifteen single nucleotide polymorphisms (SNPs) of corin gene (rs12641823, rs2271037, rs6834933, rs2271036, rs78911825, rs2351783, rs10049713, rs10517195, rs17654278, rs3749584, rs4695253, rs36090894, rs6823184, rs12509275, rs73814824) were selected using the National Center for Biotechnology Information database (http://www.ncbi.nlm.nih.gov/projects/SNP) and the Genome Variation Server database (http://gvs.gs.washington.edu/GVS147/) as previously reported.^5^, ^7^, ^30^ All the genotyping experiments were done by CapitalBio (CapitalBio Corp., Beijing, China).

### 2.5 Statistical analyses

The quality control of parental SNP data, including genotyping call rate, Mendelian consistency, MAF and HWE, was performed with PLINK software (version 1.9, http://zzz.bwh.harvard.edu/plink/). We used PLINK software to test the association of single marker with phenotypes, and three genetic models (additive, dominant and recessive) were also assumed for each SNP analysis by mixed‐effect regression models. Models were adjusted for the fixed effects of baseline age, sex, and BMI, and the random effect of familial correlations. Bonferroni correction was used for adjustment of multiple testing.

For the analyses of the incidence of hypertension, 51 participants already diagnosed with hypertension at baseline were excluded. We examined the additive association between each SNP and incidence of hypertension using a generalized linear mixed model, which permits multilevel modeling when the response variable follows a binary distribution (eg, incident hypertension). Changes in age, sex, and BMI as fixed effects and familial correlation as a random effect were adjusted in the multivariable analysis by using *glmer* function in *lme4* R package.

Gene‐based analysis was used to evaluate the overall association of a candidate gene with longitudinal BP changes and hypertension incidence, which was published and internal validity was assessed.^5^, ^7^, ^32^, ^33^ Bivariate analysis was performed to examine overall genetic association with longitudinal BP changes and hypertension incidence followed by a multivariate analysis. The truncated product method (TPM), which combines *p* values from single SNP association analyses, Gene‐based analysis was performed using *R* software (version 3.0.1; http://www.r‐project.org).

## 3 RESULT

### 3.1 Baseline characteristics and BP response to dietary intervention

Baseline characteristics and BP responses to low‐and high‐salt diets in the original cohort of Baoji Salt‐Sensitive Study (N = 514) were presented in Table 1. Overall, BP paralleled salt intake, decreased on a low‐salt diet and increased on a high‐salt diet. The BP changes during the dietary interventions were greater in probands than in their siblings, offspring, and spouses.

**TABLE 1:** Baseline characteristics and BP response to low‐and‐high salt diets

	Probands	Siblings	Spouses	Offspring	Parents
No. of participants	99	167	18	49	181
Age (years)	41.8 ± 8.4	39.8 ± 7.4	47.4 ± 6.1	23.3 ± 6.9	66.1 ± 8.3
Male (%)	69.7	49.1	26.3	49.0	48.4
Body mass index (kg/m^2^)	23.0 ± 2.8	22.2 ± 2.9	23.1 ± 4.7	20.1 ± 2.7	20.4 ± 2.6
BP at baseline (mmHg)					
SBP	120.9 ± 12.5*	107.6 ± 11.1	108.6 ± 12.2	102.7 ± 10.7	123.2 ± 21.3
DBP	79.0 ± 8.3*	70.1 ± 8.1	70.6 ± 6.9	63.4 ± 8.9	70.5 ± 10.5
MAP	93.0 ± 9.0*	82.6 ± 8.7	83.3 ± 7.9	76.5 ± 9.2	88.0 ± 13.1
BP response to low salt intervention (mmHg)					
SBP	111.7 ± 10.0* ^,^ ^&^	103.4 ± 9.1^&^	102.5 ± 7.7^&^	100.3 ± 9.4^&^	–
DBP	72.8 ± 9.3* ^,^ ^&^	66.4 ± 7.7^&^	67.1 ± 5.8^&^	60.7 ± 8.3^&^	–
MAP	85.7 ± 9.0* ^,^ ^&^	78.7 ± 7.6^&^	78.9 ± 5.4^&^	73.9 ± 8.3^&^	–
SBP change	−8.65 ± 9.52*	−3.90 ± 5.41	−6.15 ± 7.88	−2.38 ± 4.79	–
DBP change	−6.00 ± 6.71*	−3.64 ± 4.83	−3.48 ± 6.36	−2.70 ± 5.21	–
MAP change	−6.88 ± 7.07*	−3.73 ± 4.55	−4.37 ± 6.52	−2.59 ± 4.56	–
BP response to high salt intervention (mmHg)					
SBP	118.9 ± 11.2* ^,^ ^#^	108.5 ± 11.1^#^	108.4 ± 10.9^#^	102.0 ± 10.0^#^	–
DBP	76.2 ± 8.1* ^,^ ^#^	68.7 ± 9.3^#^	68.6 ± 7.5	60.9 ± 8.3	–
MAP	90.4 ± 8.5* ^,^ ^#^	82.0 ± 9.5^#^	81.9 ± 8.0^#^	74.6 ± 8.4	–
SBP change	7.16 ± 7.40*	5.09 ± 6.50	5.93 ± 7.90	1.72 ± 4.07	–
DBP change	3.49 ± 7.33*	2.29 ± 5.73	1.51 ± 4.69	0.22 ± 4.52	–
MAP change	4.71 ± 6.86*	3.22 ± 5.60	2.98 ± 5.61	0.72 ± 3.79	–

Continuous variables are expressed as mean ± SD. BP, blood pressure; SBP, systolic blood pressure; DBP, diastolic blood pressure; MAP, mean arterial pressure.

^&^ *p* < .05 vs. the baseline levels.

^#^ *p *< .05 vs. the low‐salt intervention.

* *p *< .05 vs. the siblings, spouses, or offspring.

As shown in Supplemental Table S1, the urinary sodium excretion significantly decreased from baseline to the low‐salt diet, but increased from the low salt to high‐salt diet (*p* < .05), which indicated that the subjects’ compliance with the dietary intervention protocol were excellent.

### 3.2 Corin and BP response to dietary intervention

The genome location, MAF, HWE and potential function prediction of each of the SNPs were presented in Supplemental Table S2. No SNPs deviated statistically significantly from Hardy‐Weinberg equilibrium, except for SNP rs10049713, which was excluded from the further analysis.

The associations of corin SNPs with the BP response to dietary intervention (after correcting for multiple testing) are displayed in Table 2. SNP rs3749584 was significantly associated with DBP and MAP response to low‐salt diet, while rs4695253, rs17654278 were associated with PP response to low‐salt diet. SNPs rs4695253, rs12509275, rs2351783, rs2271036, rs2271037 were significantly associated with SBP, DBP, and MAP responses to high‐salt diet.

**TABLE 2:** Associations of corin SNPs with BP response to dietary intervention

		SBP response		DBP response		MAP response		PP response	
SNP	Allele	*β*	*p*	*β*	*p*	*β*	*p*	*β*	*p*
Low‐salt intervention									
rs4695253	T	−0.044	.707	0.177	.133	0.093	.403	−0.244	.036^a^
rs12641823	A	−0.059	.435	−0.084	.256	−0.081	.277	0.009	.911
rs3749584	G	−0.127	.124	−0.349	.007^b^	−0.286	.026^b^	0.081	.455
rs6834933	C	0.018	.850	−0.023	.811	−0.007	.938	0.048	.619
rs12509275	G	−0.056	.644	0.123	.308	0.058	.633	−0.204	.092
rs2351783	T	0.027	.804	0.190	.080	0.137	.207	−0.162	.137
rs2271036	T	0.023	.782	−0.017	.838	−0.001	.987	0.048	.560
rs2271037	T	0.034	.684	−0.010	.899	0.007	.929	0.056	.497
rs10517195	G	0.006	.958	−0.003	.980	0.001	.995	0.011	.923
rs17654278	A	0.143	.094	0.017	.844	0.072	.398	0.174	.042^a^
rs36090894	A	−0.080	.408	−0.034	.722	−0.056	.556	−0.071	.462
rs78911825	C	−0.051	.812	−0.069	.744	−0.067	.751	0.003	.988
rs73814824	A	−0.041	.628	−0.017	.840	−0.029	.734	−0.037	.662
rs6823184	C	−0.025	.753	0.071	.375	0.036	.651	−0.108	.176
High‐salt intervention									
rs4695253	T	−0.273	.022^a^	−0.396	.001^a^	−0.379	.001^a^	0.118	.323
rs12641823	A	−0.107	.182	−0.079	.318	−0.095	.231	−0.049	.529
rs3749584	G	0.031	.782	0.013	.220	0.104	.342	−0.123	.266
rs6834933	C	0.053	.589	−0.050	.598	−0.014	.880	0.134	.168
rs12509275	G	−0.247	.046^a^	−0.330	.006^a^	−0.322	.008^a^	0.071	.565
rs2351783	T	−0.239	.031^a^	−0.250	.021^a^	−0.264	.015^a^	−0.017	.878
rs2271036	T	−0.180	.029^a^	−0.165	.041^a^	−0.183	.024^a^	−0.042	.614
rs2271037	T	−0.209	.012^a^	−0.195	.017^a^	−0.215	.009^a^	−0.044	.597
rs10517195	G	−0.180	.114	−0.038	.736	−0.095	.399	−0.199	.080
rs17654278	A	−0.126	.145	−0.015	.858	−0.059	.493	−0.153	.076
rs36090894	A	0.129	.188	0.049	.614	0.083	.392	0.117	.232
rs78911825	C	−0.258	.236	−0.048	.826	−0.132	.543	−0.294	.179
rs73814824	A	−0.079	.372	−0.004	.964	−0.033	.707	−0.104	.863
rs6823184	C	0.025	.762	0.002	.982	0.011	.894	0.031	.670

For associations those were not significant under any model, *β* and *P* values for an additive model are listed. All genetic models are based on the minor allele of each SNP. BP, blood pressure; SBP, systolic blood pressure; DBP, diastolic blood pressure; MAP, mean arterial pressure; PP, pulse pressure; SNP, single nucleotide polymorphism. ^a^ dominant model; ^b^ recessive model; ^c^ additive model.

### 3.3 Association analyses for longitudinal BP changes and incidence of hypertension

Table 3 summarizes the characteristics of the subjects at baseline (2004) and at follow‐up (2009 and 2012). During 8‐year follow‐up, the mean SBP, DBP and MAP increased by 14.4, 6.6, and 9.1 mmHg, respectively, and 103 (28.9%) subjects developed hypertension.

**TABLE 3:** Characteristics of the study participants at baseline and during the follow‐ups

Characteristics	Baseline in 2004	Follow‐up in 2009	Follow‐up in 2012
Gender (M/F)	267/247	208/204	185/171
Age (years)	48.6 ± 19.8	53.3 ± 14.2	56.6 ± 19.0
Body mass index (kg/m^2^)	22.2 ± 3.1	22.4 ± 3.3	23.6 ± 3.5
SBP (mmHg)	115.2 ± 17.6	120.0 ± 17.3	129.6 ± 18.7
DBP (mmHg)	71.3 ± 10.0	75.8 ± 10.4	77.9 ± 10.9
MAP (mmHg)	86.0 ± 11.5	90.5 ± 11.7	95.1 ± 11.9
Fasting glucose (mg/dL)	86.9 (80.9‐94.4)	91.5 (86.0‐99.1)	92.6 (86.7‐100.8)
Triglycerides (mg/dL)	112.7 (82.9‐158.5)	129.3 (94.5‐175.5)	119.0 (87.0‐167.4)
Total cholesterol (mg/dL)	155.5 (138.5‐177.6)	157.7 ± 29.0	162.4 (145.7‐186.4)
High‐density lipoprotein (mg/dL)	47.4 ± 11.2	50.9 ± 11.6	49.9 (42.7‐58.6)
Hypertension at baseline (n, %)	51 (9.9)	–	–
Hypertension incidence (n, %)*	–	77 (18.9)	103 (28.9)

SBP, systolic blood pressure; DBP, diastolic blood pressure; MAP, mean arterial pressure.

* Participants with hypertension at baseline were excluded. Non‐normally distributed variables are expressed as the median (interquartile range). All other values are expressed as mean ± SD or n, %.

The associations of individual SNPs in the corin gene with 5‐year (2004‐2009) and 8‐year (2004‐2012) BP changes are shown in Table 4. SNPs rs12641823, rs6834933, rs2271036 and rs22710367 were significantly associated with the changes in SBP, DBP, MAP or PP at both follow‐ups. SNPs rs3749584 and rs36090894 were significantly associated with 5‐year changes in SBP and PP. In addition, gene‐based analysis showed that corin gene was significantly associated with the longitudinal DBP change (*p_TPM_* = .0181) and MAP change (*p_TPM_* = .0099) in bivariate analysis, and remained significant in multivariate analysis (*p_TPM_* = .0224 for DBP change; *p_TPM_* = .0139 for MAP change) during 8‐years follow‐up.

**TABLE 4:** Associations of corin SNPs with BP changes from baseline to follow‐ups

SNP	BP (2004‐2009)				BP (2004‐2012)			
	SBP change	DBP change	MAP change	PP change	SBP change	DBP change	MAP change	PP change
rs4695253	0.330	0.464	0.361	0.444	0.444	0.322	0.332	0.805
rs12641823	0.005^b^	0.029^b^	0.007^b^	0.031^b^	0.019^b^	0.005^b^	0.004^b^	0.812
rs3749584	0.010^a^	0.480	0.089	0.003^a^	0.194	0.611	0.339	0.184
rs6834933	0.030^a^	0.023^a^	0.016^a^	0.231	0.011^a^	0.016^a^	0.007^a^	0.141
rs12509275	0.334	0.502	0.381	0.422	0.635	0.416	0.472	0.998
rs2351783	0.412	0.754	0.552	0.375	0.854	0.984	0.936	0.795
rs2271036	0.398	0.037^a^	0.103	0.632	0.045^a^	0.012^a^	0.013^a^	0.474
rs2271037	0.362	0.036^a^	0.094	0.690	0.051	0.012^a^	0.013^a^	0.513
rs10517195	0.055	0.638	0.979	0.295	0.543	0.184	0.275	0.821
rs17654278	0.596	0.328	0.403	0.959	0.933	0.742	0.812	0.882
rs36090894	0.082	0.767	0.502	0.020^c^	0.582	0.742	0.637	0.632
rs78911825	0.781	0.218	0.393	0.540	0.178	0.285	0.190	0.343
rs73814824	0.469	0.891	0.773	0.267	0.229	0.419	0.280	0.332
rs6823184	0.525	0.632	0.550	0.617	0.974	0.993	0.991	0.961

For associations that were not significant under any model, *P* values for an additive model are listed. All genetic models are based on the minor allele of each SNP. BP, blood pressure; SBP, systolic blood pressure; DBP, diastolic blood pressure. MAP, mean arterial pressure; PP, pulse pressure; SNP, single nucleotide polymorphism.

^a^ additive model.

^b^ dominant model.

The associations of corin SNPs with the incidence of hypertension after 5‐year (2004‐2009), 8‐year (2004‐2012) follow‐ups are presented in Table 5. SNP rs73814824 was significantly associated with the hypertension incidence over 8 years. Furthermore, gene‐based analysis showed that corin gene was significantly associated with hypertension incidence over 8‐year follow‐up (*p*_TPM_ = .0351) after multiple adjustments.

**TABLE 5:** Associations of individual SNP with hypertension incidence

SNP	Incident hypertension (2004–2009)		Incident hypertension (2004–2012)	
	*OR* (95% CI)	*p*	*OR* (95% CI)	*p*
rs4695253	−0.12 (−0.72–0.44)	.685	−0.02 (−0.53–0.47)	.924
rs12641823	−0.00 (−0.38–0.37)	.991	−0.11 (−0.44–0.22)	.514
rs3749584	0.11 (−0.44–0.63)	.682	−0.01 (−0.49–0.46)	.977
rs6834933	−0.43 (−0.97–0.07)	.104	−0.18 (−0.63–0.25)	.406
rs12509275	−0.21 (−0.83–0.37)	.496	−0.05 (−0.57–0.46)	.846
rs2351783	0.05 (−0.48–0.55)	.858	−0.08 (−0.55–0.37)	.722
rs2271036	−0.16 (−0.57–0.23)	.421	−0.09 (−0.44–0.25)	.604
rs2271037	−0.17 (−0.57–0.23)	.416	−0.08 (−0.44–0.27)	.640
rs10517195	0.24 (−0.29–0.75)	.352	0.14 (−0.33–0.61)	.555
rs17654278	−0.30 (−0.72–0.10)	.154	−0.05 (−0.41–0.29)	.741
rs36090894	0.14 (−0.36–0.62)	.575	0.14 (−0.29–0.57)	.513
rs78911825	−0.05 (−1.12–0.88)	.916	−0.28 (−1.22–0.59)	.546
rs73814824	−0.20 (−0.57–0.15)	.267	−0.48 (−0.91–−0.07)	.026
rs6823184	0.13 (−0.25–0.51)	.499	−0.03 (−0.37–0.31)	.872

SNP, single nucleotide polymorphism; OR, odds ratio; CI, confidence interval.

## 4 DISCUSSION

In this study, we identified several novel corin SNPs that were significantly associated with longitudinal BP changes and hypertension incidence. The gene‐based analyses also showed that croin gene was significantly associated with longitudinal BP changes and the incidence of hypertension. To our knowledge, this is the first study investigating such associations in humans. These findings highlight potentially important contributions of corin gene to long‐term BP regulation. Moreover, it adds to our understanding of the genetic architecture of BP progression and hypertension.

Previous animal studies indicated that corin was implicated in BP regulation and the development of hypertension. Chan and associates^10^ demonstrated that corin knockout mice had increased BP compared with wild‐type one. Atrial natriuretic peptide (ANP) is a cardiac hormone that regulates BP. They further found that corin knockout mice failed to produce mature ANP, exhibiting hypertension and cardiac hypertrophy.^10^ In addition, the associations of genetic variants in the corin gene with hypertension were also reported in different populations. Chen and associates^34^ showed that corin SNPs rs2271037 and rs3749585 were associated with increased risk of hypertension in a Han population in China. Dries and associates^15^ determined that the minor corin T555I / Q568P allele was independently associated with increased risk of hypertension in three different cohorts (Dallas heart study, Multi‐Ethnic study of Atherosclerosis, Chicago Genetics of Hypertension Study). In addition, Zhang and associates ^18^ also reported an insertional variant of corin, c.102_103insA, which occurs preferentially in hypertensive patients in China.

To the best of our knowledge, this is the first study to investigate the associations of corin gene with longitudinal BP changes and hypertension incidence over time. We found that SNPs rs12641823, rs6834933, rs2271036, and rs2271037 were significantly associated with longitudinal BP changes after 8 years of follow‐up. In addition, SNP rs73814824 was significantly associated with hypertension incidence over 8 years. Corin gene was aggregately associated with longitudinal BP changes and the incidence of hypertension in bivariate analysis and multivariate analyses. Our study provides direct evidence that corin may be involved in long‐term BP regulation and hypertension, although its genetic and physiological mechanisms remain elusive. Wang and associates ^11^ found the natriuretic peptide processing activity of the recombinant corin variant T555I /Q568P was impaired, indicating that the genetic variation of corin gene may reduce the corin activity in vivo, leading to hypertension in blacks. The association between corin gene risk variants and susceptibility to hypertension was found to be caused by abnormal natriuretic peptide processing activity, pointing to corin as a therapeutic target for hypertension.

In addition, this is the first study exploring the associations of corin genetic variants with BP responses to dietary salt intake in humans. In this study, we showed that corin SNP rs3749584 was significantly associated with DBP and MAP response to low‐salt diet, while rs4695253, rs17654278 were associated with PP response to low‐salt diet. SNPs rs4695253, rs12509275, rs2351783, rs2271036, rs2271037 were significantly associated with SBP, DBP and MAP responses to high‐salt diet. The mechanism why different SNPs were associated with different BP phenotypes is not clear and deserves further investigation. DBP reflects, to a greater extent, the trend of arterial resistances and MAP. SBP is more closely linked to variations in pulse BP and is produced by a group of factors including left ventricular ejection and the reflection of the sphygmic wave.^35^ Therefore, different SNPs may exert different effects on different stages of BP formation, and associated with different BP phenotypes. Our results provide evidence to support the hypothesis that corin genetic variations are involved in salt‐sensitivity of BP. This relationship had been confirmed by previous animal studies. Transgenic mice expressing T555I/Q568P variant in corin gene had high levels of pro‐ANP in the heart and developed salt‐sensitive hypertension and cardiac hypertrophy.^11^ Wang and associates ^12^ also showed that BP levels in corin knockout mice on a high‐salt diet was significantly increased; however, there was no such change was observed in wild‐type mice. It is interesting to speculate how corin affects salt sensitivity by regulating natriuretic peptide system. ANP pathway plays an important role in regulating BP by inhibiting aquaporin 2 (AQP2) and β‐ epithelial Na^+^ channel (β‐ENaC).^36^ Polzin and associates ^37^ showed that renal ENaC expression was increased in corin knockout mice, indicating that corin may downregulate renal ENaC expression and activity. Furthermore, Zhao and associates ^38^ suggested that ANP‐mediated inhibition of sodium reabsorption in distal nephron segments was essential to promote natriuresis. In addition, high‐salt intake induced an increase in extracellular fluid (ECF). BP may increase because of increased cardiac output and volume‐dependent factors.^39^, ^40^ As the ECF increased, corin may be activated. Therefore, corin may affect Na^+^ homeostasis or ECF and salt sensitivity of BP through these pathways. Future functional studies are needed to elucidate how the identified risk loci contribute to salt sensitivity of BP at the molecular and cellular level.

The current study has several strengths. First, participation in the dietary interventions was high, and excellent compliance with the study interventions was noted, as evidenced by 24‐h urinary sodium excretions during each intervention period. Furthermore, stringent quality control procedures were employed for genotyping and data collection. We used the average of nine separate BP measures at baseline and each follow‐up examination, thus reducing measurement errors. However, this study also has some limitations. Firstly, BP measurement with a mercury sphygmomanometer may cause confounding effect of the white coat phenomena. The study population was relatively small and restricted to northern Chinese individuals. Therefore, the novel findings in our study need to be replicated in other cohorts with different genetic backgrounds. In addition, no washout period was inserted between low‐and high salt diets, which may influence the final results. However, the dietary intervention protocol we adopted was based on The Genetic Epidemiology Network of Salt Sensitivity (GenSalt) study, which was widely used by other researchers.^41^, ^42^, ^43^, ^44^ Finally, due to the limited number of genotyped SNPs in corin gene, less frequent genetic variants may have been omitted in the current study.

In conclusion, we report for the first time that corin gene polymorphisms were significantly associated with BP response to dietary salt intervention in Chinese Han population. Using single‐marker and gene‐based analyses, this study further provides direct evidence for the role of corin gene in longitudinal BP phenotypes and hypertension incidence. The findings from the current study provide a basis for potential prevention and a possible therapeutic target for hypertension in the future. In addition, this work contributes to a cumulative understanding of the genomic mechanisms that regulate BP and the development of hypertension.

## CONFLICTS OF INTEREST

The authors declare that there is no conflict of interest.

## AUTHOR CONTRIBUTIONS

Yang Wang and Jian‐Jun Mu contributed to research conception and design. Jian‐Jun Mu recruited subjects. Zou Ting, Ming‐Fei Du, Chao Chu, Gui‐Lin Hu, Yue‐Yuan Liao, Chen Chen, Dan Wang, Qiong Ma, Yu Yan, Hao Jia, Ke‐Ke Wang, Yue Sun, Ze‐Jiaxin Niu, Xi Zhang, Rui‐Chen Yan, Zi‐Yue Man, Dan‐Feng Ren, Lan Wang, Wei‐Hua Gao, Hao Li, Yong‐Xing Wu, Chun‐Hua Li, Ke Gao, and Jie Zhang performed experiments. Ting Zou, Shi Yao, and Tie‐Lin Yang analyzed data. Ting Zou and Yang Wang drafted manuscript. Jian‐Jun Mu and Yang Wang edited and revised manuscript. All authors read, critically revised, and approved the final version of manuscript.

## Supporting information

Supporting information
