# Supplementary material for: Associations of corin genetic polymorphisms with salt sensitivity, blood pressure changes, and hypertension incidence in Chinese adults
Source: J Clin Hypertens (Greenwich). 2021 Nov 30;23(12):2115–23. doi: 10.1111/jch.14401 (PMC8696215; doi:10.1111/jch.14401)
Supplement: Supplementary file 1 — Supporting information [file JCH-23-2115-s001.doc]

**Supplemental Table S1**. Effects of dietary intervention on urinary sodium and potassium excretions

|  | **Probands** | **Siblings** | **Spouses** | **Offspring** |
| --- | --- | --- | --- | --- |
| Baseline | | | | |
| 24 h Urinary sodium, mmol | 225.1±11.6 | 213.8±16.4 | 218.3±20.8 | 205.4±23.4 |
| 24 h Urinary potassium, mmol | 36.8±10.8 | 38.1±9.13 | 39.4±12.3 | 35.8±15.7 |
| Low-salt intervention | | | | |
| 24 h Urinary sodium, mmol | 54.9±11.3 | 53.6±9.8 | 52.8±13.5 | 58.2±15.1 |
| 24 h Urinary potassium, mmol | 35.4±8.9 | 39.8±7.6 | 34.2±6.7 | 40.5±9.8 |
| High-salt intervention | | | | |
| 24 h Urinary sodium, mmol | 317.0±21.8 | 304.4±28.6 | 318.6±20.5 | 298.4±25.1 |
| 24 h Urinary potassium, mmol | 43.4±13.9 | 39.6±9.1 | 40.2±8.3 | 41.1±14.5 |

Continuous variables are expressed as mean ± SD.

**Supplemental Table S2.** Information on genotyped SNPs of corin gene

| **SNP** | **Region** | | **Position** | **Alleles *^a^*** | **MAF** | ***P*-value*^b^*^†^** | **Potential function prediction** |
| --- | --- | --- | --- | --- | --- | --- | --- |
| rs12641823 | | 47596708 | UTR3 | A/G | 0.429 | 0.338 | — |
| rs2271037 | | 47680085 | intronic | T/G | 0.401 | 1 | — |
| rs6834933 | | 47604134 | intronic | C/A | 0.231 | 0.163 | — |
| rs2271036 | | 47679928 | intronic | T/C | 0.402 | 1 | — |
| rs78911825 | | 47757453 | intronic | C/T | 0.034 | 1 | — |
| rs2351783 | | 47669315 | intronic | T/C | 0.169 | 0.116 | DHS |
| rs10049713 | | 47822814 | intronic | C/T | 0.163 | 9.89E-05 | — |
| rs10517195 | | 47682174 | exonic | G/A | 0.156 | 0.258 | — |
| rs17654278 | | 47693816 | intronic | A/G | 0.315 | 0.728 | TFBS; DHS |
| rs3749584 | | 47597654 | UTR3 | G/C | 0.137 | 0.773 | — |
| rs4695253 | | 47596632 | UTR3 | T/C | 0.137 | 0.746 | — |
| rs36090894 | | 47710982 | intronic | A/G | 0.197 | 0.501 | TFBS |
| rs6823184 | | 47839013 | intronic | C/T | 0.349 | 0.517 | TFBS; DHS |
| rs12509275 | | 47629866 | intronic | G/T | 0.130 | 0.315 | — |
| rs73814824 | | 47779024 | intronic | A/G | 0.354 | 0.312 | — |

SNP, single nucleotide polymorphism; MAF, minor allele frequency. TFBS, transcription factor binding site; DHS, DNase I hypersensitive site. parents only (parental generation).*^b^P* values of Hardy-Weinberg equilibrium test. *^a^* Alleles are presented as major: minor allele.
